# The impact of mobility scooters on their users. Does their usage help or hinder?: A state of the art review

**DOI:** 10.1016/j.jth.2015.03.005

**Published:** 2015-06

**Authors:** Roselle Thoreau

**Affiliations:** The Accessibility Research Group, University College London, Department of Civil and Environmental Engineering, University College London, Gower Street, London WC1E6BT, UK

**Keywords:** Mobility scooters, Transport, Health, Mobility, Physical activity

## Abstract

As older people start to have difficulty in walking many choose to use a mobility scooter to help them move around. Benefitting from improved design, mobility scooters are becoming an increasingly popular mobility device and are a common sight on many streets. However, very little is known about their usage or their impact in terms of either quality of life or functional health. Whilst mobility scooters may help to improve the quality of life of their users, it is also possible that the sedentary nature of their usage results in a decline of physical functionality and therefore reduced capabilities. Before any substantial research can be carried out it is crucial to understand the importance of a mobility scooter on the lives of the people that use them and to review the initial research published on the effect of scooter use on physical health. This paper is a state-of-the-art review. It describes the current research knowledge on mobility scooters, shows where gaps in knowledge exist and where future research needs to focus.

## Highlights

• Research literature surrounding mobility scooters is sparse.
• Scooter users feel their scooter has a positive impact on their life.
• Scooter training is seen as important but this does not always occur.
• The impacts of scooter use on health, in the short term, are not detrimental.
• The long-term links between scooter usage and physical functionality is unclear.

## 1 Introduction

Every year every person in England makes an average of 923 journeys, 22% of these are by foot (National Travel Survey, 2014). The health outcomes of active transport, such as walking are widely acknowledged (Carsperson and Fulton, 2008; Hamer and Chida, 2007; Lee and Buchner, 2008; Murtagh et al., 2010). Many older people have difficulty in walking and the percentage of people in this group rises with age (Mindell and Craig, 2005). Depending on the reasons behind the difficulty, as people begin to struggle to walk they have a range of options open to them which can be used alone or in combination. They can; walk less often, walk less far, take more frequent rest breaks while walking, use public or private transport, use a mobility aid for stability such as a walker or a cane, or use a mobility device instead of walking such as a wheelchair or a mobility scooter.

Mobility scooters are becoming an increasingly common sight on many streets. Benefitting from improved design and image as well as a decrease in usage stigma, mobility scooters have become an increasingly popular mobility aid. They can be hired in large supermarkets, in shopping centres, at some tourist attractions and visitor centres and are widely available for purchase including on the high street. However, despite their prevalence little is known about their impact upon their users physical health and physical capabilities.

From a health literature perspective a mobility scooter can be seen as a walking and physical activity replacement. It enables its user to travel distances they previously would have made by foot (or short distance vehicle trips) without any physical effort (Hoenig et al., 2007; Steyn and Chan, 2008; Zagol and Krasuski, 2010). For some older people a mobility scooter can be a replacement for a car and for the types of trips they would have made with a car. However a mobility scooter also has the potential to replace shorter trips that car drivers might previously have considered too short to drive, and therefore would have walked. For an older adult with difficultly maintaining their previous levels of walking, using a mobility scooter allows them to participate in activities they previously could not access, to participate in activities without discomfort or to extend the duration of participation.

The evidence supporting the health benefits of physical activity for older adults is well documented (Ferrucci et al., 2004; Guralnik et al., 1993; Grossman and Stewart, 2007; Manson et al., 2002; Taylor et al., 2004). On the one hand, the mobility scooter, as a sedentary mobility device may plays a detrimental role in the health of its user. On the other hand, the popularity of the device suggests that there are great benefits to its use. It is important to understand the role mobility scooters plays in older people physical health so that we can ensure older people who use scooters get the greatest benefits without risking their future physical functionality. Before any substantial research can be carried out to untangle the complexity of the impact mobility scooter usage has on physical health it is crucial to understand the importance of a mobility scooter on the lives of the people that use them and to review the initial research published on the effect of scooter use on physical health.

This paper is a state-of-the-art review of the current literature available. It examines where knowledge gaps lie and where future research is and should be focussed.

## 2 Background

Mobility scooters are a single occupant electronic transport vehicle and are used as a mobility aid. A solely battery operated device; it usually has between three and five wheels and is steered using a handlebar. Different scooters can be ridden either on the pavement or the road depending on speed capability and they may include a horn, lights and space for storage. They are often referred to as power-operated vehicle/scooters or electric scooters (May et al., 2010; Steyn and Chan, 2008). Mobility scooters are designed for and used by individuals who are able to walk and manipulate themselves on and off a seated object. Unlike wheelchairs, mobility scooters are generally treated as vehicles in the sense that they do not have to be guaranteed access into buildings. This means that in order to access services and activities users must be able to walk.

In the United Kingdom (UK) there are numerous ways to access mobility scooters. Many retail outlets sell them, including a major high street seller, specialist retails stores and multiple online providers. Additionally, they are bought second-hand. Many loan schemes for mobility scooters exist. Although the National Health Service (NHS) does not provide patients with scooters some local councils, for example Camden Council (2014) operates a long-term loan scheme and short-term hire schemes. Some large supermarkets loan scooters to shoppers free of charge while they are on the premises. The largest scheme giving access to mobility scooters in the UK is Shopmobility. Shopmobility is a lending scheme based in shopping areas who lend mobility scooters, powered wheelchairs and manual wheelchairs to people whilst they are in the shopping district (Gant, 2002). Charging for use varies but most schemes are free. The service is offered to anyone who is permanently or temporarily disabled though no proof is required making the scheme essentially available to be used by anyone. Users must become members of the scheme and training on usage is offered at this stage. Once a member bookings can be made in advance of arrival.

Laws regarding scooters in the UK are set out by the Department for Transport (2012). Scooters are defined as class two or class three vehicles. No driving licence is required to operate them. Both classes of vehicle must be driven by people who are disabled and are 14 or older. However it is not clear whether these rules are being enforced in class two mobility scooters (Barton et al., 2014). Class three vehicles must be registered with the Driver and Vehicle Licensing Agency (DVLA), although they do not pay road tax they do have to display a NIL tax disk. Class two scooters are those that cannot exceed 6.44 km/h (4 mph), can be used on the pavement and cannot be used in the road except where crossing it. Class three scooters can travel up to 12.9 km/h (8 miles). They are allowed by law on the road if they are travelling at greater than 6.44 km/h but must not travel on motorways. Class three vehicles must have lights, mirrors and a horn.

The mobility scooter is considered to be an assistive technology. Assistive technology is defined by the World Health Organisation (2004) as any device or system that allows individuals to perform tasks that they would otherwise be unable to do or increases the ease and safety with which tasks can be performed. In order to carry out the desired activities, for example visiting family or going shopping, users need the physical functionality mentioned above. Safe operation of the scooter also requires the user to be able to turn their head to look behind them, although class three scooters, and some class two scooters include rear view mirrors. Safe operation also requires the ability to balance when the scooter is driving on a slope, on rough grounds or on and off pavements. Despite the necessary physical functionality when moving on and off the mobility scooter, the actual operation is a mainly passive task, requiring only a minimal amount of grip strength to engage the accelerator. In this sense the scooter does not assist the user to walk but removes the necessity of the task (at least while on the scooter).

In the United Kingdom, mobility scooters are an entirely optional device. A mobility scooter has not been designed, nor has built the environment infrastructure been altered to allow for mobility scooters to access most buildings. Therefore, a person using a mobility scooter needs to be able to walk albeit for short distances and/or with assistance. Whereas wheelchairs, electric or manual, are provided by the National Health Service (2014) (NHS), the choice to use a mobility scooter is made by the individual. Mobility scooters are bought privately, although a registered disabled person can apply for a small subsidy (Motability, 2014).

## 3 Methodology

This review examines the current knowledge of mobility scooters in relation to the effects they have on the user, in particular the user perspective of their device and any changes to the physical health of the user. In this case physical health is referring more specifically to physical functionality of mobility in the users over time, i.e., the maintenance of their capabilities of walking at the level they could before they began to use a scooter. Emphasis has been placed on older people, as one of the most visible users (Barton et al., 2014) and the group of people more likely to become frail (Rockwood et al., 1999). Older people is a term which can range in meaning from all those above 60 years old to an older subset of this group or simply those of pensionable age (Gilleard and Higgs, 2011; Roebuck, 1979; United Nations, 2002; Victor, 2010). Some studies make no mention of age, where the focus is on the injury, disability or capability of the user.

In gathering the evidence this paper includes papers and reports with a variety of research designs, including both larger controlled trials and smaller case studies, using either qualitative or quantitative methods. Literature was identified by searching electronic databases, SCOPUS, PubMed, PsychINFO, EMBASE and AMED. The search terms used were: mobility scooters, electric scooters, motoris(z)ed scooters, and powered mobility devices. The reference lists of relevant papers were examined to locate any secondary sources not gathered through the original search. Government websites (Department for Transport, Office for National Statistics and Department of Health) were searched for relevant statistics, reports or policy documents. The criteria for inclusion was (1) primary source studies, (2) studies involving adults (3) studies which included outcomes for mobility scooter users as separate from other personal mobility devices and (4) studies presented in English. The articles were then filtered to remove irrelevant papers (for example, papers on childrens push scooters and mopeds). No papers were found on accidents involving scooters with the exception of media publications which were not included as they recounted singular accidents with little objective evidence.

Literature on mobility scooters can be divided into three categories, (1) prevalence within the population, (2) user perspectives and (3) physical functionality and physical capability impact.

## 4 Discussion

There is a dearth of literature on mobility scooters. Where it does exist it is often research in combination with and undifferentiated from electric wheelchairs. Like scooter users, many manual wheelchair users have some physical functionality that allows them some mobility (Hoenig et al., 2002). However, whilst a useful starting point the evidence from these studies will not always be relevant to scooter users. In the UK electric wheelchairs are only provided on the NHS to those people who need wheelchairs fulltime and are unable to propel themselves in a manual wheelchair (Standards for Better Health, 2005) unlike a mobility scooter, which is a private purchase. To gain a wider understanding of what may be relevant to mobility scooter users, some evidence on wheelchairs have been included here. Where evidence relates only to scooters this has been made apparent.

### 4.1 Prevalence

There have been many attempts to quantify the number of mobility devices, particularly wheelchairs, in different countries. This data would be useful to help to understand the population who use them and to follow any trends in prevalence and their impact. Due to limited registration requirements and a lack of clear differentiation between mobility scooters and wheelchairs, accurate numbers are not available (Barton et al., 2014). However, some estimates of numbers and evidence of trends do exist.

#### 4.1.1 Wheelchairs

The number of wheelchair users in the UK has increased. Evidence has been found that between 1986 and 1996 the number of wheelchair users doubled (Manty et al., 2007). Current figures for wheelchair use in England are estimated at 1.2 million, with 825,000 of those being regular, long term users (Huonker et al., 1998; Papworth Trust, 2010).

#### 4.1.2 Mobility scooters

Mobility scooter numbers are less well documented than wheelchair numbers (Barton et al., 2014). Where documented they reflect wheelchairs in their increasing numbers. In 2009 the sales of mobility scooters in the UK totalled £83 million but this had increased to £96 million in 2013 (Keynote Ltd, 2014). This rise is reflected in global figures of £182 million in 2009 rising to an estimated £245 million in 2013. Projected estimates for 2017 global sales reaches £335 million (Global Industry Analysts, 2012). In 2006 it was estimated that around 25,000 mobility scooters were bought each year in the UK (Barham et al., 2006) and it is now estimated that approximately 80,000 are being bought each year (Barton et al., 2014). An estimated 350,000 are currently being used in the UK (Barton et al., 2014). Ricability׳s survey found that 47% of their mobility scooter respondents were over 65 (Barton et al., 2014), lower than the estimated 74% of wheelchair users over 60 (Sapey et al., 2004). Using the estimate by Barton et al. and ONS (2013) population data, percentages of users can be calculated. 1.5% of the population over 65 uses scooters compared with 0.5% in the general population. This percentage is similar to Thoreau (2011) who used the English Longitudinal Study of Ageing database (ELSA) to discover the proportion of over 65 year olds who use mobility scooters. ELSA included a question on mobility scooter use (rather than ownership). Thoreau (2011) examined a subset of ELSA and found 1.4% of those aged over 65 used a mobility scooter.

Whilst the use of mobility devices including mobility scooters is increasing there is no evidence that the number of people with difficulty walking has increased. For example, US research shows that the number of people unable to walk 400 m (quarter of a mile) has not changed over time (Auger et al., 2008; LaPlante, 2003). LaPlantes data is from across all ages and they state that the data does not shown clearly whether increases in mobility device use is down to older people or non older people. It has been suggested that the growth in usage is down to a decrease in usage stigma and improved device image and design (LaPlante, 2003).

### 4.2 User perspectives

Studies on user perspective or user experiences are crucial to ensuring mobility scooters meet their users requirements and highlight where improvements can be made. Whilst studies on different aspects of user perspective exist on assistive technology devices only two studies have focussed solely on mobility scooters and their users (Barton et al., 2014; May et al., 2010).

#### 4.2.1 Person-device match

The unregulated access to assistive mobility devices including mobility scooters, while giving potential users freedom of choice, does have a disadvantage. It means a lack of good advice to assess the suitability of a device to a person and vice versa. There is a great need for an assistive technology device to match an individual׳s capability/mobility needs (National Health Service, 2011). When a device is matched correctly the device is seen by the individual as empowering and giving them more freedom. When the device does not suit them users lack confidence and are at higher risk to their own safety (Bergen, 1997).

#### 4.2.2 Training and guidance

The amount of training given to users influences their likelihood to use the device (National Institute of Health and Clinical Excellence, 2006). Whilst there is support for training for safe use (Mortenson et al., 2014; Townsend and Watson, 2013) training does not always occur. Estimates of the number of scooter users who receive training vary widely. An international survey of scooter users found only 25% had received training (Mortenson et al., 2014). However, a UK study found that a majority of users, 59%, received training, with 42% of users receiving the training from the organisation they bought their scooter from (Barton et al., 2014). A focus group of scooter users and stakeholders recognised that there were safety risks involved in using scooters but there is no data to prove this (Barton et al., 2014). Training does occur but is not available at a national level. Local schemes are often run by the police (for example Norfolk Police (2014) run training events), or mobility centres (for example Parkgate Mobility (2014), run a scheme in Yorkshire).

Only a third of wheelchair and mobility scooter users ask for guidance from a health professional before buying their device (Bowling and Stenner 2011). In the UK some advice is available. Disability Rights UK, a disability network, provides an online guide to the range of scooters available and some guidance on how to choose the right one for individual needs (Campbell, 2014). Ricability, a consumer research charity, creates independent reports for older and disabled people on various assistive technology goods. They have a guide on using mobility scooters on public transport and choosing the right scooter (Ricability, 2014; Jacobs et al., 2013). The Department for Transport (2012) also offers some advice on choosing a suitable mobility scooter as well as explanations on legal rules and requirements.

#### 4.2.3 Satisfaction, independence and wellbeing

Research around user satisfaction, independence and wellbeing specifically of mobility scooters is sparse. With the exceptions of two papers (May et al., 2010 and Barton et al., 2014), any research on mobility scooters in these areas is combined and undifferentiated with electric wheelchairs.

Studies of a range of assistive mobility devices for mobility found that users felt their device enabled them to participate in more activities, gave them greater independence and increased their sense of security (Brandt et al., 2004; Evans et al., 2007; National Health Service, 2010; Ordonez, 2006; Woods and Watson, 2003; Wressle and Samuelsson, 2004). Evidence specifically from mobility scooters show that users generally view their devices positively, associating them with the freedom to move independently outside the house, in some cases being housebound without them (May et al., 2010).

A small study of powered wheelchair and scooter users (Sammuelsson and Wressle, 2014) found a high level of satisfaction and ease in activity participation after uptake of their devices. Users found that their ability to socialise, be mobile and their sense of safety, independence and self-esteem all raised as a result of device uptake. The studies findings are limited by its small sample size (20 mobility scooter users and 4 electric wheelchair users) and its lack of differentiation in its results between the different types of user by device. However, given that 80% of the sample are scooter users it can be concluded that scooter users do gain satisfaction, security and independence from using their scooter.

Barton et al. (2014) surveyed a total of 480 mobility scooter users of all ages in the UK. It is the first large survey of scooter users in the UK. The survey gives some useful insight into scooter user satisfaction and travel behaviour. This was a self-selecting sample of users, the majority of whom, 88%, owned their own scooter. Respondents were asked why they chose to use a mobility scooter, instead of a wheelchair. The two most common responses were that scooters were easier to use (61%) and that scooters were more comfortable (52%). In addition they found that users relied on their scooter to get around, with 74% of respondents saying they would not make the same trips without their scooter. Of those who felt they could make the same trips without the mobility scooter only 10% felt they could make the trip by walking.

May et al. (2010) surveyed a total of 66 scooter users and held focus groups with an additional 15 users. The research focussed on users experiences with their scooters and gathered data only from users over 65. Users started using scooters to maintain their levels of mobility either as a result of losing physical capabilities or when they stopped driving. Users satisfaction with scooter comes from users enhanced mobility. Respondents felt that using a scooter meant they were able to travel to more destinations, achieve more daily tasks, maintain more independence and increase their sense of wellbeing.

Both these two studies provide insight into the experiences of scooter usage. May et al. is particularly useful in understanding the experiences of older users. Both studies show that users view their scooters as a very positive part of their lifestyle. The main negative aspects to their experience are from a lack of accessibility from the built environment. By using current scooter users the data gathered is likely to be positively skewed. For a more rounded understanding it would be illuminating to talk to people who potentially could use scooters but do not and people who have used scooters in the past but no longer do.

Negative views of devices stem from accessibility and from interaction with pedestrians (Brandt et al., 2004; Evans et al., 2007; Steyn and Chan, 2008; May et al., 2010). In a study of different mobility devices, dissatisfaction was recorded where users found their device limited where they could access (Evans et al., 2007). In their study of mobility scooter users and powered wheelchair users, Brandt et al. (2004) also noted that some users had encountered difficulties in carrying out their activities and that the older the users were the less they felt their device was suitable for the activities they wanted to complete. These findings are echoed by mobility scooter users who found that accessibility into buildings, along pavements and on sloped surfaces was limited (Barton et al., 2014; May et al., 2010; Edwards and McCluskey, 2010).

#### 4.2.4 Activity

Common activities carried out using mobility scooters were: going for a ride, shopping, daytrips and social visits to family or friends (Barton et al., 2014; Brandt et al., 2004; Edwards and McCluskey, 2010; May et al., 2010). Scooter trips are typically made by users between three to five times per week (May et al., 2010) and the most common activity to carry out using a scooter was shopping, followed by visiting local places (Barton et al., 2014).

Two studies (Brandt et al., 2004; May et al., 2010) found evidence suggesting that use of both powered wheelchairs and mobility scooters should be taken up earlier and be used by people who were less impaired than the study sample so as to delay activity dropout levels as a result of immobility (the Brandt et al. study covered mobility scooters and powered wheelchairs whereas May et al. study only looked at mobility scooters). This conclusion fits well with satisfaction literature, but its advice works against the literature on physical functioning and sedentary lifestyles.

### 4.3 Physical health

The bulk of the research in this area has been carried out on wheelchair users, their physical activity levels and their physical functionality and physical capabilities. This research is based on those who use their wheelchairs on a full time basis (generally Spinal Cord Injury patients, e.g., Haisma et al., 2006). There are two reasons that these findings cannot be generalised to scooter users. Firstly, scooter users are able to walk, albeit often for only a limited distance. Secondly, manually propelled wheelchairs require physical effort to propel their chairs forward whereas scooter users do not (Suzuki et al., 2012). The research in this section is focused only on those studies that separately examine mobility scooters.

There are opposing views regarding the use of assistive technology which is physically passive and their impact upon physical functionality that can apply to mobility devices such as mobility scooters (Hoenig et al., 2007; Steyn and Chan, 2008; Weiss et al., 2007). On the one hand it is possible that mobility device use, including mobility scooter use, increases participation in both physical and social activities outside the home that users would have been unable to participate in without using such a mobility aid (Brandt et al., 2004; May et al., 2010; Ordonez, 2006; Woods and Watson, 2003). Access to these activities, via mobility aid use, may increase aspects of quality of life and wellbeing in users (Steyn and Chan, 2008; May et al., 2010). On the other hand assistive technology devices that are completely passive when the user does have some physical function, run the risk of de-conditioning the users physical functionality and their mobile capabilities at a faster rate than if they had used a more physically active assistive technology (Weiss et al. 2007). It has been argued that scooters are a lifestyle choice rather than a medical necessity (Hendry and McVittie, 2004) and therefore there is value in considering whether this lifestyle choice could be harming long term physical capabilities. Aside from theorising only two studies, Hoenig et al., 2007 and Zagol and Krasuski, 2010, have focused on objective functional physical health measures and mobility scooter use.

Hoenig et al. (2007) study aimed to understand the effect of scooter use on the walking ability of people with knee osteoarthritis or rheumatoid arthritis. This randomised control study involved participants with either condition, who were able to walk 15 m independently. Participants were randomly either given scooters or maintained their usual care (control group). Participants walking abilities were tested, using a 6-min walk test, one month and three months after the scooter group began to use mobility scooters. Participants were questioned on the type of activities they participated in during the time period. The study found no significant differences in scooter users walking abilities when compared to the control group. However, scooters users were found to participate in a wider range of activities when they used the mobility scooters. The study concluded that, in terms of walking ability, mobility scooter use creates no adverse effects.

The randomised control methodology means the results will be accurate despite the small sample size (*n*=16). However, this study has a number of limitations. First, the study revisits the participants after 3 months and can only provide evidence for short term effects. This evidence cannot be used to understand or predict the effect over a longer period of time. It can be surmised that most scooter users will use their scooters for a longer time period than 3 months (Barton et al. found that most users have owned their scooters for at least two years) and this length of time might be too short to pick up evidence of a change in locomotory capabilities. It would have been more interesting had the group been studied over a longer period to determine the existence, timing, and persistence of any such change. Secondly, the study examines individuals with a specific condition known to affect mobility. From this viewpoint the study can make no comment on those who take up scooters for other reasons (for example as a result of pre-clinical disability). Thirdly, the scooter group were more likely to already be using wheelchairs at baseline. If these users are merely substituting time spent in the wheelchair with time spent in the scooter then no extra sedentary behaviour is occurring and therefore minimising the effects.

Zagol and Krasuski (2010) aimed to understand whether providing patients with mobility scooters increased their cardiovascular risk. The study was a retrospective analysis of data of patients from an army medical centre in the United States. Patients who had received a mobility scooter within a six-year period (1998–2004) were included and their medical data one-year prior and one year post receiving their scooter was extracted (*n*=102). Once selected, this group was sent a questionnaire on usage of the mobility scooter, as well as perceived wellbeing and quality of life post and prior to receiving a scooter.

From the data available, BMI, weight, cholesterol, blood pressure, fasting glucose level and medication was included. This enabled a cardiovascular risk to be created for each individual. Cardiovascular risk was measured for 12 months before a mobility scooter was prescribed, as a baseline, and 12 months after a mobility scooter was prescribed.

The study found a statistically significant increase in fasting glucose level (from 119–133 mg/dl), in haemoglobin Alc (6.3 to 6.8) and in the incidence of diabetes. BMI did not change and nor did systolic blood pressure. However, 20% of patients had their blood pressure medication increased or had additional blood pressure medication prescribed during this time. At odds with the medical data, the questionnaire data found that patients felt their mental wellbeing, their perceived physical functionality and their overall quality of life had improved between pre and post mobility scooter uptake. The results of this study provide some evidence that mobility scooter use may have negative impacts on physical functionality.

The study had a couple of limitations. Firstly, no control group was studied and it is therefore it is not known whether a similar population without scooters would have similar changes in cardiovascular risk. A matched control group from the same database would have shown whether or not this was the case. In response to this criticism from Hoenig et al. (2010), Zagol and Krasuski stated that the incidence of diabetes in their sample was much higher than expected in an age-adjusted population (9.1 in 1000 individuals in the United States versus 301.6 per 1000 individuals in the sample). Secondly, as it is impossible to isolate all the overlaying factors the changes must be acknowledged as a correlation and causality cannot be assumed.

### 4.4 Policy

In the UK there has been some policy interest in mobility scooters. The governments House of Commons Transport Committee (2009–2010) focussed their attention on safety regulations and reports of accidents, noting anecdotal evidence of increases in numbers of users. It was recommended that any future legislations must carefully worded so not to deprive users of their only independent transport mode. The Department for Transport commissioned Ricability to carry out a study on the practices and policies related to scooter use on public transport (Jacobs et al., 2013). The study identified a lack of information about mobility scooter specifications and recommended that more information needed to be made available to allow transport operators to know which scooter types would fit on their vehicles and for users to know which operators allowed scooters on-board and what dimensions and permits were required.

## 5 Current and future research

There are many aspects of mobility scooter use that would be useful to explore. Given the evident upward trend of the use of mobility scooters this is crucial to understanding the role mobility scooters can play in individuals׳ lives. Currently the impact of mobility scooters on their users could be detrimental or beneficial in a variety of different ways. Could and should they be medically prescribed (they can be claimed on medical insurance in America but not in the UK)? Should they be guaranteed to be accommodated in public transport or in public buildings? Without a comprehensive body of research neither individual users, carers, health professionals or policy makers are able to make informed decisions on their use in a way that would be beneficial.

Older people are the group most likely to develop mobility difficulties and the most likely to start using a mobility scooter. Research, undertaken by the Accessibility Research Group at University College London is currently investigating the impact that mobility scooter use has on long term health in older people. This research is a longitudinal study using quantitative and qualitative data from mobility scooter users and non-mobility scooter users. Prior to mobility scooter uptake both groups had similar levels of physical capabilities. A scoping study using ELSA data of scooter users over 65years old has concluded that mobility scooters users perform worse at physical functioning tasks than other old people (Thoreau, 2011). Additionally, mobility scooter users have the highest rates of non-completion of physical tasks due to incapacity. The reasons behind the low scores and declines in capability are unclear and cannot be unearthed using the currently available data. However, scooter users poor record shown here indicates the need for it to be investigated, something which the follow up research will achieve. To balance the research on physical functionality further investigation into the psychological gains such as on independence and wellbeing is being undertaken. Results from these studies is expected in late 2015.

## 6 Conclusions

Research literature and empirical studies surrounding mobility scooters are sparse. In terms of user experience most users felt their scooter has had a positive impact upon their lives and perceive their scooter in a positive light. Their scooter meets their needs by enabling them to independently achieve their desired activities. It is clear that matching the mobility device to the individual and training the individual to use their mobility device is important. However, neither of these occur regularly.

The impacts of scooter usage on functional health is less clear. The relationships between frequency/length of use to physical functionality and capabilities has not been investigated. Where mobility scooter data does exist it is most often inseparable from wheelchair data, particularly electronic wheelchair data. Given the different physical capabilities of their users this is unhelpful. The two works that focus solely on mobility scooters and physical health impacts investigate different aspects of physical health (physical and functional), have different limitations and reach different conclusions.

Health research into mobility scooters is underexplored. Physical health literature is clear that a lack of physical exercise leads to a loss of functional capabilities including mobility in older adults. Also known is that the use of wheelchairs and scooters is increasing in the population despite no increases in levels of people with mobility difficulties (Aijanseppa et al., 2005; Auger, 2008; LaPlante, 2003). What is not clear is the role that mobility devices, particularly those where no physical effort is required, play. Whilst there is a wealth of data on the relationships between physical activity, health and ageing there is a lack of evidence on the role mobility devices play in promoting physical functioning and physical capabilities. The debate between mobility scooter users positive experiences and perceptions and the possibility that using scooters causes functional decline is of great interest and importance. As these topics have had such little quantification there is value in amassing evidence for both topics and how they interact. It is plausible that some scooter users sacrifice physical functioning for improved activity participation and independence. Empirical evidence showing the benefits and disadvantages of scooter usage is needed to allow those prescribing, recommending or choosing to use a scooter to make a fully informed choice.

## References

[bib1] Aijanseppa S., Notkola I.L., Tijhuis M., van Staveren W., Kromhout D., Nissinen A. (2005). Physical functioning in elderly Europeans: 10 year changes in the north and south: the HALE project. J. Epidemiol. Community Health.

[bib2] Auger C., Demers L., Gelinas I., Jutai J.W., Noreau L. (2008). Power mobility for middle-aged and older adults. Am. J. Phys. Med. Rehabil..

[bib3] Barham P., Fereday D., Oxley P. (2006). Review of Class 2 and Class 3 Powered Wheelchairs and Powered Scooter (Invalid Carriages).

[bib4] Barton, C., Holmes, J. Jacobs, C., 2014. Mobility scooters: a market study. Research Institute for Consumer Affairs. 〈http://www.rica.org.uk/sites/default/files/documents/pdfs/research-consultancy/Rica%20Mobility%20scooter%20market%20study%20final.pdf〉 (accessed 8.12.14.).

[bib5] Bergen A. (1997). Mobility: taking control of life. Except. Parent.

[bib6] Bowling A., Stenner P. (2011). Which measure of quality of life performs best in older age? A comparison of the OPQOL, CASP-19 and WHOQOL-OLD. J. Epidemiol. Community Health.

[bib7] Brandt A., Iwarsson S., Stahle A. (2004). Older people׳s use of powered wheelchairs for activity and participation. J. Rehabil. Med..

[bib8] Campbell, D., 2014. Get Mobile. Disability Rights, London. 〈http://disabilityrightsuk.org/sites/default/files/pdf/getmobile.pdf〉 (accessed 8.12.14.).

[bib9] Camden Council, 2014. 〈http://www.camden.gov.uk/scootability〉 (accessed 8.12.14.).

[bib10] Carsperson C.J., Fulton J.E. (2008). Epidemiology of walking and type 2 diabetes. Med. Sci. Sports Exerc..

[bib11] Department for Transport, 2012. Mobility scooters and powered wheelchairs on the road: some guidance for users. Department for Transport, London. 〈https://www.gov.uk/government/uploads/system/uploads/attachment_data/file/211030/advice-for-mobility-vehicle-users.pdf〉 (accessed 8.12.14.).

[bib12] Edwards K., McCluskey A. (2010). A survey of adult power wheelchair and scooter users. Disabil. Rehabil. Assist. Technol..

[bib13] Evans S., Frank A.O., Neophytou C., de Souza L. (2007). Older adults use of, and satisfaction with, electric powered indoor/outdoor wheelchairs. Age Ageing.

[bib14] Ferrucci L., Guralnik J.M., Studenski S., Fried L.P., Cutler G.B., Walston J.D. (2004). Designing randomized, controlled trials aimed at preventing delaying functional decline and disability in frail, older persons: a consensus report. Am. Geriatr. Soc..

[bib15] Gant R. (2002). Shopmobility at the millennium ‘Enabling’ access in town centres. J. Transp. Geogr..

[bib16] Gilleard C., Higgs P. (2011). Frailty, disability and old age: a re-appraisal. Health.

[bib17] Global Industry Analysts, Inc. 2012. Wheelchairs (powered and manual): a global strategic business report. 〈http://www.strategyr.com/Wheelchairs_Powered_and_Manual_Market_Report.asp〉

[bib18] Grossman M., Stewart A. (2007). You aren׳t going to get better by just sitting around. Am. J. Geriatr. Cardiol..

[bib19] Guralnik J., LaCroix A.Z., Abbott R.D., Berkman L.F., Satterfield S., Evans D.A., Wallace R.B. (1993). Maintaining mobility in late life. I. Demographic characteristics and chronic conditions. Am. J. Epidemiol..

[bib20] Haisma J.A., van der Woude L.H.V., Stam H.J., Bergen M.P., Sluis T.A.R., Bussmann B.J. (2006). Physical capacity in wheelchair-dependent persons with a spinal cord injury: a critical review of the literature. Spinal Cord.

[bib21] Hamer M., Chida Y. (2007). Walking and primary prevention: a meta-analysis of prospective cohort studies. Br. J. Sports Med..

[bib22] Hendry F., McVittie C. (2004). Is quality of life a healthy concept? Measuring and understanding life experiences of older people. Qual. Health Res..

[bib23] Hoenig H., Pieper C.F., Zolkewitz M., Schenkman M., Branch L.G. (2002). Wheelchair users are not necessarily wheelchair bound. J.Am. Geriatr. Soc..

[bib24] Hoenig H., Pieper C.F., Branch L.G., Cohen H.J. (2007). Effect of motorized scooters on physical performance and mobility: a randomized clinical trial. Arch. Phys. Med. Rehabil..

[bib25] Hoenig H., Huffman K., Lezzoni L., Levy C.E., Sonnenblum S., Sprigle S. (2010). Motorized scooters: boon or bane?. Am. J. Cardiol..

[bib26] House of Commons Transport Committee, 2009–2010. Mobility scooters. 〈http://www.publications.parliament.uk/pa/cm200910/cmselect/cmtran/414/414.pdf〉 (accessed 8.12.14.).

[bib27] Huonker M., Schmid A., Sorichter S., Schmidt-Trucksab A., Mrosek P., Keul J. (1998). Cardiovascular differences between sedentary and wheelchair-trained subjects with paraplegia. Me. Sci. Sports Exerc..

[bib28] Jacobs, C., Barton, C., Harnett, M., 2013. The Carriage of Mobility Scooters on Public Transport. Research Institute for Consumer Affairs (Rica). 〈http://www.rica.org.uk/sites/default/files/documents/pdfs/research-consultancy/dft-mobility-scooter-research-final-report-april-2013.pdf〉 (accessed 8.12.14.).

[bib29] Key Note Ltd, 2014. Equipment for the disabled market update 2014. 〈http://www.keynote.co.uk/market-intelligence/view/toc/product/10926/equipment-for-the-disabled/2/contents?medium=toc#〉

[bib30] LaPlante M.P. (2003). Demographics of Wheeled Mobility Device Users. Space Requirements for Wheeled Mobility.

[bib31] Lee I.M., Buchner D.M. (2008). The importance of walking to public health. Med. Sci. Sports Exerc..

[bib32] Manson J.E., Greenland P., LaCroix A.Z., Stefanick M.L., Mouton C.P., Oberman A., Perri M.G., Sheps D.S., Pettinger M.B., Siscovick D.S. (2002). Walking compared with vigorous exercise for the prevention of cardiovascular events in women. N. Engl. J. Med..

[bib33] Manty M., Heinonen A., Leinonen R., Tomakangas T., Sakari-Rantala R., Hirvensalo M., vonBonsdorff M.B., Rantanen T. (2007). Construct and predictive validity of a self-reported measure of preclinical mobility limitation. Arch. Phys. Med. Rehabil..

[bib34] May E., Garrett R., Ballantyne A. (2010). Being mobile: electric mobility-scooters and their use by older people. Ageing Soc..

[bib35] Mindell J., Craig R. (2005). Health Survey for England.

[bib36] Mortenson W.B., Hoag E., Higgins R., Emery R., Joyce L. (2014). Stakeholders׳ perspectives related to the development of a scooter training program. Disability and Rehabilitation: Assistive Technology.

[bib37] Motability Operations Limited, 2014. 〈http://www.motability.co.uk〉 (accessed 8.12.14.).

[bib38] Murtagh E.M., Murphy M.H., Boone-Heinonen J. (2010). Walking – the first steps in cardiovascular disease prevention. Curr. Opin. Cardiol..

[bib39] National Health Service (2010). Statistics on obesity, physical activity and diet: England, 2010.

[bib40] National Health Service (2011). Physical activity guidelines for older adults (65+ years).

[bib41] National Health Service, 2014. National wheelchair managers forum. Directory of Services. 〈http://www.wheelchairmanagers.nhs.uk/services.html〉

[bib42] National Institute for Health and Clinical Excellence (2006). Obesity: the prevention, identification, assessment and management of overweight and obesity in adults and children.

[bib43] National Travel Survey, 2014. National travel survey: England 2013. Department for Transport. 〈https://www.gov.uk/government/uploads/system/uploads/attachment_data/file/342160/nts2013-01.pdf〉

[bib44] Norfolk Police, 2014. SafeScoot. 〈http://www.norfolk.police.uk/safetyadvice/safescoot.aspx〉 (accessed 8.12.14.).

[bib45] Office of National Statistics, 2013. Population Estimates for U.K., England and Wales, Scotland and Northern Ireland, mid 2011 and mid 2012. 〈http://www.ons.gov.uk/ons/publications/re-reference-tables.html?edition=tcm%3A77-319259〉 (accessed 3.3.15.).

[bib46] Ordonez J. (2006). It׳s the new walking. Scooters evolve from medical need to lifestyle choice. Newsweek.

[bib47] Papworth Trust, 2010. Facts and figures. Disability in the United Kingdom 2010. 〈http://www.papworth.org.uk/downloads/disabilityfactsandfigures2010_100202152740.pdf〉.

[bib48] Parkgate Mobility, 2014. Scooter safe. 〈http://www.parkgatemobility.co.uk/scootersafe-mobility-scooter-training/〉 (accessed 8.12.14.).

[bib49] Ricability, 2014. 〈http://www.rica.org.uk/content/choosing-mobility-scooter〉 (accessed 8.12.14.).

[bib50] Roebuck J. (1979). When does old age begin?: The evolution of the English definition. J. Soc. Hist..

[bib51] Rockwood K., Stadnyk K., MacKnight C., McDowell I., Hébert R., Hogan D.B. (1999). A brief clinical instrument to classify frailty in elderly people. Lancet.

[bib52] Sammuelsson K., Wressle E. (2014). Powered wheelchair and scooters for outdoor mobility: a pilot study on costs and benefits. Disabil. Rehabil. Assist. Technol..

[bib53] Sapey B., Stewart J., Donaldson G. (2004). The Social Implications of Increases in Wheelchair Use.

[bib54] Standards for Better Health, 2005. Health care standards for wheelchair services under the NHS. Version 005. 〈http://www.wheelchairmanagers.nhs.uk/servicestandards.pdf〉

[bib55] Steyn P.V., Chan A.S. (2008). Mobility Scooter Research Project.

[bib56] Suzuki, T., Uchiyama, H., Holloway, C., Tyler, N., 2012. Assisting control for attendant propelled wheelchair based on force velocity relationship. Engineering in Medicine and Biology Society (EMBC), In: Proceedings of the IEEE 2012 Annual International Conference. 10.1109/EMBC.2012.6346613 23366574

[bib57] Taylor A., Cable N.T., Faulkner G., Hillsdon M., Narici M., Van Der Bij A.K. (2004). Physical activity and older adults: a review of health benefits and the effectiveness of interventions. J. Sports Sci..

[bib58] Thoreau R.J. (2011). Personal Mobility Scooters: Health differences between mobility scooter users and the unaided pedestrian.

[bib59] Townsend K., Watson A. (2013). Competent use of a motorised scooter—assessment, training and ongoing monitoring: a vital role for occupational therapy practice. Aust. Occup. Ther. J..

[bib60] United Nations, 2002. Department of Economic and Social Affairs. World Population Ageing 1950–2050. 〈http://www.un.org/esa/population/publications/worldageing19502050/pdf/95annexi.pdf〉 (accessed 8.12.14.).

[bib61] Victor C. (2010). Ageing, Health and Care.

[bib62] Weiss C., Hoenig H., Fried L.P. (2007). Compensatory strategies used by older adults facing mobility disability. Arch. Phys. Med. Rehabil..

[bib63] World Health Organisation (2004). A Glossary of Terms for Community Health Care and Services for Older Persons.

[bib64] Woods B., Watson N. (2003). A short history of powered wheelchairs. Assist. Technol..

[bib65] Wressle E., Samuelsson K. (2004). User satisfaction with mobility assistive devices. Scand. J. Occup. Ther..

[bib66] Zagol B.W., Krasuski R.A. (2010). Effect of motorized scooters on quality of life and cardiovascular risk. Am. J. Cardiol..

